# Knowledge and attitude of children about soda and its effects on dental health

**DOI:** 10.6026/973206300181016

**Published:** 2022-10-31

**Authors:** Siraj DAA Khan, Bassam Hassan Al Qahtani, Hamad Mohammad Alamer, Mohammed Saleh Al Dashil, Ziyad Mohammed Asiri, Yasir Ali Asiri, Mansoor Hamad Alkorbi, Abdullelah Abduaslam Altouk

**Affiliations:** 1 Department of Preventive Dental Sciences, Faculty of Dentistry, Najran University, KSA

**Keywords:** Consumption, carbonated beverages, attitude, practice, prevention

## Abstract

The consumption of carbonated beverages or soda drinks has increased due to media advertisements. These drinks have ill effects on dental as well as on general health especially in children and adolescent. Therefore, a study was carried out to check the knowledge and attitude of children in Saudi Arabia. A questionnaire-based study was conducted. It comprises of three parts i) demographic details ii) attitude toward consumption of soda drinks and iii) knowledge about soda drinks. A total of 393 primary elementary School Children age range 5 to 15 years old responded the questionnaire. 47.3% children drank soda on weekly basis. More than half participants did not have knowledge about the effects of soda on health (55.2%) and on teeth (53.9%). The knowledge of children about the effects of soda drinks on dental health in not convincing one. However, their attitude was relatively better. There is a need to improve their practices for prevention of dental and general health.

## Background:

In dental science, demineralization has become significant in the last few years and many technologies have been used for its treatment. [1, 2] Tooth erosion is mainly caused due to many factors that increase dental abnormalities in young and old patients. The most important dental factors are lifestyle factors, diet, gastric acid, vomiting or gastro esophageal reflux disease, and fruit-flavored beverages. [3, 4] While, eating too much sweets and junk food also cause tooth erosion in the most severe cases. [5] Therefore, other factors are related to acidic drinks that causes too much acidity. [2] Energy drinks are main cause of dental erosion, which leads to increase the beverage markets about the exceeding 5.8 billion liters in 160 countries in 2013. [6] Such variety of drinks also useful for providing energy due to the presence of caffeine, taurine, and sugar content. [6, 7] The consumption of energy drinks is greater among young individuals due to their excellent properties such as taste and flavouring that also leads to boost energy in some way. [7] Among of soft drinks, most of them have acidic nature. [4, 8] Daily use of soft and energy drinks has severe effects on both dental and general health especially it cause dental caries and potential use of artificial sweetened soft drinks also cause the complications associated with dental erosion. [9] Dental degradation is caused by lots of factors. Among of them, long-term intake of carbonated soft drinks that prolonged the risk of dental erosion. [10] These carbonated beverages are usually manufactured by adding the specific amount of different kinds of food acids sweetener, and sugar. [11] Recent clinical and experimental studies showed that continuous consumption of these soft drinks damage the surface of dental and therefore causing the dental deterioration. [12, 13] Daily consumption of NCSD (non-carbonated soft drinks) in excessive amount has several effects on health as well as metabolism such as increasing the high blood sugar, cancer and diabetes. [14, 15] These drinks especially affect the health of children with low immunity and old patients underlying chemotherapy. [16] While, NCSD in the exceeding amount have several effects on health that disrupt the metabolism in young and old patients. [17] Dental science is highly effective for the treatment of dental-based diseases and its preventive measures. Higher consumption of SSBs (sugar-sweetened beverages) causes metabolic syndromes, poor oral health and type 2 diabetes. [18] Development of lethal type of type-2 diabetes and weight gain related to the consumption of added. [19] Modern health style is one of the risks for dental erosion and damaging the dental surface among today’s adolescents. [20] Skudutyte-Rysstad et al. [21] conducted the clinical study to establish the relationship between erosive wear and daily consumption of acidic beverages that increased the damage to dental surfaces and between erosive wear and gender in Norwegian 18-year-olds. In a Swedish study among adolescents aged 13-19 years, Hasselkvist et al. [9] it has been reported that inadequate daily use of soft drinks and unhealthy lifestyle have lethal effects on dental health causing the borne of different diseases such as erosive lesions and caries (DMTF/DMTS). Different dental health professionals have adapted strategies in order to overcome the risks factors associated with health and dental caries for healthy teeth. Different dental-based clinical studies have been extensively carried out about use of soft drinks worldwide but research in Saudi Arabia is not satisfying. This questionnaire-based study was designed in order to determine the level of soda and energy drinks consumption and the status about health impacts among the children of the Saudi Arabia as children health is more important and their teeth are sensitive to dental corrosion. Therefore, it is of interest to access status of carbonated drinks in the dental health of children also to evaluate the health risks and frequency of consumption of soft drinks.

## Materials & Methods:

### Study design:

The study was a questionnaire based cross-sectional. Demographic details (Age, gender, education, nationality) as well as attitude and knowledge based questions were asked from the participants.

### Setting of study:

This study was confined to the children of aged between 5-15 years old.

### Duration:

2-3 months

### Sample size and technique:

Total 393 children participated in this study. Among the participants, 120 were 5-10 years old while 273 were 10-15 years old. Convenient method for sampling was used during study duration. The purpose of the study was described to them and each question was elaborated to the participants.

### Inclusion criteria:

Children from All over Saudi Arabia aged between 5-15 years old were included. Only those participants who gave their consent were included.

### Exclusion criteria:

Children above the 15 years were not included.

## Results:

Total 393 children from age of 5 to 15 years old participated in this survey. A total number of 120 (30.5%) children were between 5-10 years old while 273 (69.5%) had age 11-15 years. As concern with the gender of the participants, 273 were males and 120 were females. A large number of children (92.1%) were Saudi National while few non-Saudi children (7.9%) also took part in this study. The education level of 160 (40.7%) children were primary and 233 (59.3%) were of elementary level. 328 (83.5%) were residents of Najran while 65 (16.5%) were not resident of Najran (Table 1 - see PDF). All responses of participants were counted in comparison of their age groups. A total number of 52 (13.2%) children from the age group of 5-10 years said they eat sweet products on weekly basis and same response was given by 110 (28.0%) participants from the age between 10-15 years. Only 5 (1.3%) and 12 (3.1%)from both age groups (5-10 years and 10-15 years respectively) respondents answered that they had never eat sweet products. When they asked about use of energy drinks, the large number of participants 73(18.6%) from 5-10 years and 125 (31.8%) from 10-15 years)said that they never used energy drinks while there are few children 6 (1.5%) from 5-10 years and 14 (3.6%)from 10-15 years) who used energy drinks3 or more times on daily basis.The usage of soda on weekly basis got the highest number of respondents, 62 (15.8%) and 124 (31.6%) from 5-10 and 10-15 years old respectively. The response was significant (P<0.05). There were 11 (2.8%) participants of aged 5-10 years and 29 (7.4%) of age 10-15 years who agreed that they drink soda 3 or more times per day. 166 (42.2%) 10-15 years old participants and 61 (15.5%) 5-10 years old children thought that the timing for drink soda is with meals. Whereas the timing of drink soda is morning was said by 7 (1.8%) any 11 (2.8%) children of age group 5-10 and 10-15 years, respectively. 27 (6.9%) 5-10 years old and 118 (30.0%) 10-15 years old participants didn’t take any special kind of soda usually. This response by participants showed significant (P<0.01) effect (Table 2 - see PDF). To evaluate the knowledge of participants about soda drinks and their effect on health different questions were asked from them. 41 (10.4%) from 1st age group (5-10 years) and 104 (26.5%) from 2nd age group (10-15 years) considered soda healthy for themselves. While 52 (13.2%) and 110 (28.0%) children from 5-10 and 10-15 year old groups denied it respectively. 2 (0.5%) participants from each group said that soda is good for food digestion. 2 (0.5%) participants from 5-10 year old and 6 (1.5%) children from age 10-15 year old agreed that soda has good taste. A large number of participants 360 (91.6%) did not answer this question. The number of participants who knew the effect of soda on health was less than 50 % (41 (10.4%) from 5-10 years and 135 (34.4%) from 10-15 years old). It should be noted that others did not have this knowledge. Only this question about knowledge of participants showed significant (P<0.01) while all other responses had non-significant effect. Tooth decay was reported the main effect by soda and energy drinks by 53 (13.5%) participants from 5-10 years old and 101 (25.7%) from 10-15 years old. 49 (12.5%) children from 1st age group (5-10 years old) and 132 (33.6%) from 2nd age group (10-15 years old) were aware about the effects of soft drinks on teeth. 30 and 63 participants from aged 5-10 and 10-15 years old respectively agreed that soft drinks cause erosion of teeth while the tooth decay was said by 58 (14.8%) and 143 (36.4%) children from 1st and 2nd age group, respectively. Most of the participants (18% from 5-10 years old and 43.3% from10-15 years old) from both age groups didn’t wash their teeth after consuming soft drinks (Table 3 - see PDF).

## Discussion:

Carbonated drinks have become today's pattern; more particularly, we can call them "fashion" among young people. The components of these drinks are sugar, acidic agents, water, CO2, caffeine, high fructose corn syrup, stabilizing and emulsifying agents and coloring agents.[22] Only a few number of studies on practice and knowledge about soda drinks have been performed in Saudi Arabia. Therefore, the present study was carried out among children of aged between 5 to 15 years. The consumption of carbonated drinks among adolescents (6-17 years old) has been increased 48%. Though the largest source to access soft drinks is home environment, however vending machines, establishment of fast food and other sources had a great share. [23] Current study revealed that 13.2% children consume soda 1-2 times per day and 47.3% drank weekly. These findings were more than another study where 21.6% drank weekly and very few participants (8.4%) consume soft drinks daily. These studies were inconsistent with another finding in which participants who drank 1-2 times and 3-4 times per day were 28.6%. [25] In present study 41.2%, children had knowledge about the effects of soda drinks on health which is in correspondence with another study where 37.71% participants were well aware about ill effects of soft drinks. [25] But these results were very less in comparison the other studies where Gupta et al. [26] and Kharde et al. [27] noted that 54% and 72.7% individuals had knowledge about the bad effects of these drinks.

Physical properties and structural integrity of teeth changes with the exposure of acidic substances that lead to the softening and ultimately loss of dental tissues.[28] Among all the soft drinks carbonated beverages are considered most dominant in which Coca-Cola taking the top place. The low price of coca cola might be a reason for its enhanced consumption. [29] About 53.9% participants of present study were aware about the effects of soda drinks on teeth. This observation is more than the findings of Mohammad et al. [25] (26.27%) and Kharde et al. [27] (10%) but less than Rai et al. [22] (98.57%).These findings were consistent with some other studies that revealed the low knowledge about ill effects of carbonated beverages on teeth. [30, 31] Negative effects of soda drinks on composite filling as well as on tooth enamel have been reported by many researchers. [32, 33] It has been demonstrated that physical properties i.e. elasticity module and hardness which results enamel erosion. In current study, tooth erosion was reported the main effect of soda drinks by 23.7% and tooth decay by 51.1%. [32] 57.8% children of present study said that the timing of soda drink is with meals while only 4.6% participants agreed on morning time. This result is contrary to the findings of Pengpid, 2020 who found that most of the participants in Malaysia consume drinks during breakfast. [34]

## Conclusions:

The knowledge of children who participated in this survey about the effects of soda drinks on dental health in not convincing one. However, their attitude was relatively better but there is a need to improve their practices for prevention of dental as well as general health. A holistic approach his recommended solving the problems that arose from the consumption of soda drinks. Change of behavior is needed in youngsters. It will help for the development of preventive and curative health program.
